# Predictors of long-term care admission in a rural and remote memory clinic

**DOI:** 10.1177/13872877261434989

**Published:** 2026-03-25

**Authors:** Anas Arwini, Andrew Kirk, Megan O’Connell, Debra Morgan

**Affiliations:** 1 College of Medicine, 12371 University of Saskatchewan, Saskatoon, Saskatchewan, Canada; 2 Division of Neurology, 7235 University of Saskatchewan, Saskatoon, Saskatchewan, Canada; 3 Department of Psychology, 7235 University of Saskatchewan, Saskatoon, Saskatchewan, Canada; 4 Canadian Centre for Rural and Agricultural Health, 7235 University of Saskatchewan, Saskatoon, Saskatchewan, Canada

**Keywords:** Alzheimer's disease, caregiving, dementia, long-term care, rural population

## Abstract

**Background:**

Caregivers face unique challenges when the care recipient transitions from at-home care to long-term care (LTC).

**Objective:**

We aimed to elucidate predictors of LTC admission within two years of initial presentation to a Rural and Remote Memory Clinic in Saskatchewan.

**Methods:**

Analysis included 635 patients seen between March 2004 and June 2019 (admitted to LTC within two years = 222, not admitted = 413). Patients were assessed neuropsychologically and administered questionnaires.

**Results:**

The majority of those who moved to LTC were diagnosed with Alzheimer's disease. The multivariate logistic regression model revealed no statistically significant results. However, univariate logistic regressions showed that advanced age (OR = 1.05, CI = 1.04–1.07), female sex (OR = 1.79, CI = 1.28–2.52), higher Functional Activities Questionnaire (OR = 1.09, CI = 1.06–1.11), lower Mini-Mental State Examination (OR = 0.861, CI = 0.827–0.897), and higher Clinical Dementia Rating score (OR = 1.13, CI = 1.06–1.21) were significant predictors (p < 0.001).

**Conclusions:**

Being older, female, more dependent in activities of daily living, and having more severe dementia predicted LTC admission, potentially helping in planning care.

## Introduction

As of 2020, approximately 600,000 Canadians were living with dementia, a number projected to reach 1 million by 2030.^1^ An estimated 350,000 caregivers each dedicate, on average, 26 h a week to informal caregiving.^1^ Dementia is an umbrella term encompassing several diseases describing the deterioration of cognitive domains and can significantly limit a person's functional capacity to carry out activities of daily living.^2^ Dementia has become a public health priority due to its rising incidence, economic cost, and significant stress to caregivers.^3^ Disease progression increases difficulty for informal caregivers (hereafter caregivers) to meet the needs of a person living with dementia in the community, heightening distress and stress.^4^ The effects of caregiving and the deteriorating cognition and independence of a person living with dementia often leads to long-term care (LTC) admission.^5–8^

Timely admission to LTC is vital for optimal care of patients and caregivers alike.^9–11^ This is particularly important as delayed or suboptimal institutionalization may fail to alleviate or even exacerbate caregiver distress.^12^ The disease progresses and cognitive decline continues, so the role of caregiver, typically spouses or family members, becomes increasingly difficult and may pose significant challenges to their caregiver. A unique stage in the caregiving journey is the time of transition to LTC. This transition is often experienced as a stressful process that brings uncertainty and distress.^13,14^ Caregivers’ distress in general leads to decreased caregiver health, significantly affecting their utilization of home-based, community-based, and institutional care services such as relocating the care recipient to LTC prematurely or to institutions that are not the best fit for them.^14^ Indeed, more attention must focus on easing the transition from caregiving to LTC admission; to facilitate this, it would be helpful to be able to predict factors associated with admission into LTC.

Previous literature has been relatively consistent on a multifactorial set of predictors of LTC admission, such as subjective caregiver stress,^5–8^^,15^ reduced functional capacity of the person living with dementia to perform activities in daily living,^5–8^^,15^ poor cognition,^6–8^^,15^ and neuropsychiatric symptoms.^6–8^ Caregiver stress and functional dependence especially were robust predictors among a sample of 2014 dyads across several European countries.^5^ These studies explored the broad, holistic experience of caregiving without clarifying which specific aspect of the caregiver experience, such as the severity of or stress arising from neuropsychiatric symptoms, is significantly contributing to LTC admission.

A common limitation in previous literature is a lack of generalizability to rural populations, who often have poorer access to healthcare services.^16,17^ Rural settings may present unique transitional challenges, requiring a tailored approach as compared to their urban counterparts.

The province of Saskatchewan lies in western Canada and has a population of 1.1 million, with roughly a third living rurally.^18,19^ Rural communities face difficulties accessing health care services, leading to the establishment of the Rural and Remote Memory Clinic (RRMC) in 2004. The RRMC aims to increase availability and accessibility of dementia services to rural Saskatchewan communities, streamlining the diagnosis, treatment plan, and counselling process, particularly for those with complex and atypical dementias.^20^ The RRMC is run by an interdisciplinary team consisting of a neurologist, neuropsychologists, physiotherapist, psychometrist, and nurse.^20^ Patients seen at the day-long initial visit are administered a standard battery of diagnostic examinations including neuropsychological assessments and questionnaires, bloodwork, brain imaging, and other investigations at the team's discretion. The goal of this process is to achieve a diagnosis and treatment plan by the end of the initial visit. Follow-up appointments, via telehealth video conference, occur at 6 weeks, 12 weeks, 6 months, then at 6-month intervals or as needed.

We aimed to determine predictors of LTC admission in persons living with dementia in rural Saskatchewan within two years of initial clinic visit. Ascertaining influencing predictors would, ideally, enable providers and caregivers to support a timely transition to long-term care and/or implement strategies to intervene and delay the need for long-term care, thereby mitigating poor outcomes related to delayed, rushed, or premature transitions to long-term care.

## Methods

Before participation, informed consent was obtained from both the patients and their caregivers. This retrospective study was conducted in accordance with the Declaration of Helsinki and was approved by the University of Saskatchewan REB (Bio #4818) on May 21st, 2024.

### Participants

**Figure 1.**
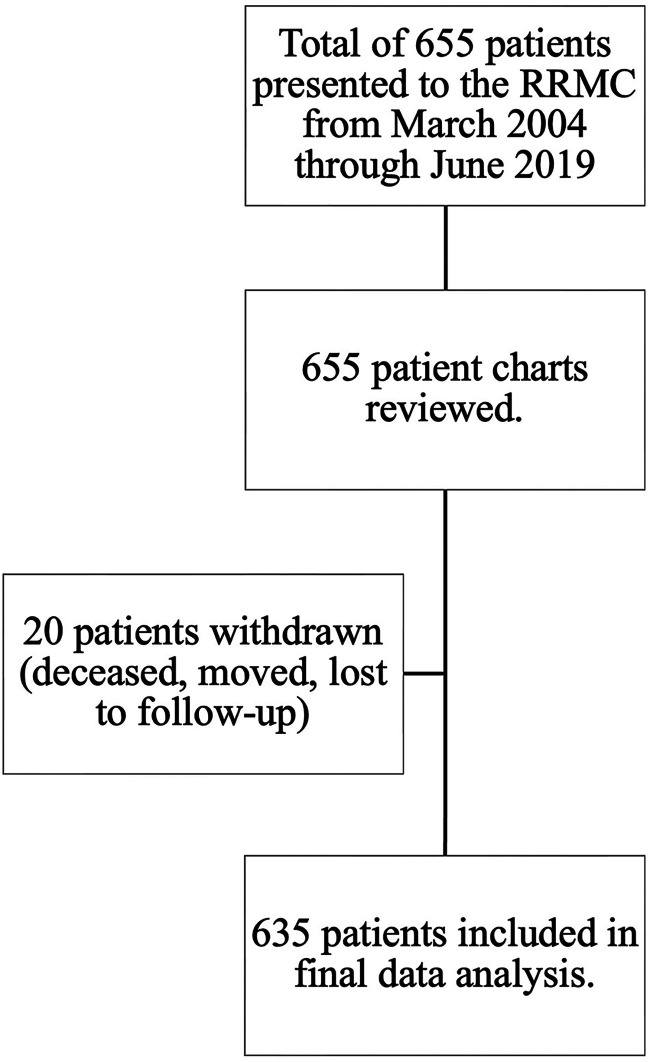
Participant flow diagram. Flow of participants throughout the study, including enrollment, chart review, and data analysis. A total of 655 patients were seen at the Rural and Remote Memory Clinic from March 2004 through June 2019. All 655 patient charts were reviewed and assessed for eligibility. Twenty patients were then withdrawn because they were deceased, had moved, or were lost to follow-up. Data from 635 participants were included in the final analysis.

A total of 655 patients were seen at the RRMC between its onset in March 2004 through June 2019. Patients were referred to the clinic by their family physicians or nurse practitioners. Inclusion criteria included those who presented to the clinic and were administered the series of assessments and questionnaires. The RRMC only accepts referrals of community-dwelling patients and so, none were in long-term care at time of assessment. 655 charts were reviewed and from those, 20 were withdrawn due to death, patients moving, or loss to follow-up within two years of first clinic visit. A final total of 635 was used for data analysis. See Figure 1 below for a participant flow-chart.

### Data collection

Data collection began in March 2004. Patients and caregivers were seen at a one-day in-person visit where each patient was interviewed by the multidisciplinary team. Patients received a standardized investigative battery including a neuropsychological assessment, bloodwork and neuroimaging, and others at the team's discretion. Both patients and caregivers completed self-report questionnaires that assessed mood, functional activities of daily living, memory, quality of life, health, and education. Caregivers completed self-report questionnaires to assess for distress. The diagnosis at initial clinic visit was collected and used for analyses.

### Measures

To evaluate the aforementioned constructs, a series of psychometrically validated scales were administered such as the Mini-Mental State Examination (MMSE), Functional Assessment Questionnaire (FAQ), Neuropsychiatric Inventory (NPI), and the Clinical Dementia Rating (CDR) scale.^21–25^ All data was then securely entered into a database.

### Assessment of cognition and dementia

The MMSE is a brief screening tool used to assess cognitive difficulties across domains such as language, registration and recall, attention, arithmetic, and orientation.^21^ It is scored out of 30, where a lower score indicates greater cognitive difficulties. The CDR scale is a global rating tool used to stage dementia across six domains, including memory, problem-solving and judgement, orientation, community affairs, personal care, and home and hobbies.^25,26^ The CDR scores each item on a scale of 0 (no impairment) to 3 (severe). Scores are then summed (termed the Sum of Boxes) for a maximum total of 18, where a higher score reflects a more severe stage of dementia.

### Assessment of functional status

The FAQ is a brief 10-item tool completed by the caregiver to assess functional status in daily activities.^23^ Each item is scored on a scale of 0 (independent) to 3 (dependent). Scores are then aggregated for a total score out of 30. A higher FAQ score indicates greater functional dependence.

### Assessment of neuropsychological symptomatology

The NPI is a comprehensive battery intended for persons with dementia that assesses frequency, severity, and distress associated with behavioral disturbances across twelve domains including ten behavioral areas and two neurovegetative areas.^24^ For this study, only the severity (NPI-S) and distress (NPI-D) scales were considered. The NPI-S is, in this case, a 12-item subscale that is scored on a scale of 1 (mild) to 3 (severe). The score is then aggregated into a severity score out of a maximum of 36. A higher score indicates more severe (i.e., more distressing to the patient and difficult to redirect) neurobehavioral disturbance. The NPI-D is a 12-item scale scored from 0 (not at all distressing) to 5 (extremely distressing). Scores are summed to a maximum possible of 60, where a higher score reflects increasing caregiver distress levels.

### Analyses

Descriptive analyses for the variables were conducted for each group. Means and variability, reported as standard deviations, were recorded for the continuous variables. Frequencies were recorded for the categorical variables. A multivariable logistic regression analysis including all predictors was conducted, followed by a series of univariate logistic regression analyses, each including a single predictor variable, to predict long-term care admission within 2 years of initial visit to the clinic (yes or no). A *p* value less than or equal to 0.05 was considered significant. Data were analyzed using IBM SPSS version 29.

## Results

### Participants

A total of 635 participants were included in data analyses. The sample included slightly more women than men. Among those who moved to LTC within two years (*n* = 222), the most frequent diagnosis was Alzheimer's disease (AD, *n* = 136) followed by non-AD dementia (*n* = 46). Of the non-AD dementias, frontotemporal dementias were the most frequent (*n* = 21) followed by Lewy body dementia (*n* = 14). All participants were accompanied by a caregiver. For most, this was their spouse/common-law partner (*n* = 104), followed by daughters (*n* = 63). Average age of participants who transitioned to LTC was 73.9 and had an average of 10.6 years of formal education. Among those not admitted to LTC (*n* = 413), the most frequent diagnosis was subjective cognitive decline (*n* = 156), followed by AD (*n* = 107). Most caregivers accompanying the participants who were not admitted to LTC were, again, their spouses/common-law (*n* = 200), followed by their daughters (*n* = 71). Participants not admitted to LTC were, on average, 67.9 years old and had 10.9 years of formal education. For a complete overview of participants’ demographic characteristics and assessment scores, see Table 1 below.

**Table 1.** Participant demographics.

		In LTC	
		Yes	No
Categorical variable		*n (%)*	*n (%)*
Gender (*n* = 646)			
	Male	73 (32.6)	196 (46.4)
	Female	151 (67.4)	226 (53.6)
Caregiver relationship to patient			
	Spouse/common-law	104 (51.0)	200 (61.9)
	Daughter/DIL	63 (30.9)	71 (22.0)
	Son/SIL	17 (8.3)	25 (7.7)
	Brother/Sister	8 (4.0)	7 (2.1)
	Friend	2 (1.0)	7 (2.2)
	Mother/Father	2 (1.0)	5 (1.5)
	Niece/Nephew	2 (1.0)	2 (0.6)
	Uncle/Aunt	0 (0)	2 (0.6)
	Grandchildren	4 (2.0)	1 (0.6)
	Formal caregiver	2 (1.0)	3 (0.9)
Frequency of contact with caregiver^a^			
	Every day	63 (82.9)	107 (82.9)
	A couple of times a week	10 (13.2)	14 (10.9)
	Once a week	0 (0)	5 (3.9)
	A couple of times a month	1 (1.3)	2 (1.6)
	Several times a year	2 (2.6)	1 (0.8)
Number of people currently living with			
	0	52 (27.7)	49 (14.5)
	1	115 (61.2)	206 (61.1)
	2	12 (6.4)	35 (10.4)
	3	6 (3.2)	23 (6.8)
	4	1 (0.5)	11 (3.3)
	5+	2 (1.0)	13 (3.9)
Diagnosis (n = 627)			
	Normal/SCI	9 (4.2)	156 (41.1)
	MCI	25 (11.6)	67 (17.6)
	Alzheimer's disease	136 (63.0)	107 (28.2)
	Non-AD dementia	46 (21.3)	50 (13.2)
		In LTC	
		Yes	No
Continuous variable		Mean ± SD	Mean ± SD
Age, in years (*n* = 667)		73.9 ± 9.10	67.9 ± 12.3
Years of formal education (*n* = 567)		10.6 ± 3.36	10.9 ± 3.51
MMSE, score between 5–30 (*n* = 562)		21.7 ± 5.04	25.1 ± 4.36
FAQ, score between 0–30 (*n* = 423)		14.2 ± 7.96	8.96 ± 7.75
NPIS, score between 1–30 (*n* = 487)		8.72 ± 6.31	7.76 ± 5.88
NPID, score between 0–60 (*n* = 508)		9.50 ± 9.44	8.33 ± 9.02
CDR-SB, score between 0–18 (*n* = 305)		6.07 ± 3.70	4.26 ± 3.79

AD: Alzheimer's disease; CDR-SB: Clinical Dementia Rating Sum of Boxes; DIL: daughter-in-law; FAQ: Functional Assessment Questionnaire; LTC: long-term care; MCI: mild cognitive impairment; MMSE: Mini-Mental Status Exam; NPID: Neuropsychiatric Inventory Distress scale; NPIS: Neuropsychiatric Inventory Severity scale; SCI: subjective cognitive impairment; SIL: son-in-law

^a^ Many responses missing because caregiver did not have the chance to complete the question.

### Multivariable logistic regression model

A multivariate logistic regression analysis was conducted to evaluate all predictors, including age, sex, education, FAQ, MMSE, NPI-S, NPI-D, CDR-SB scores can uniquely predict LTC admission. These were entered in simultaneously into the model. With this model, no predictors were statistically significant as all p-values exceeded the threshold of 0.05. This included age (OR = 1.01, 95%CI = 0.961–1.06, p = 0.717), sex (OR = 1.69, 95%CI = 0.715–4.01, p = 0.231), education (OR = 1.07, 95%CI = 0.931–1.23, p = 0.336), FAQ (OR = 0.943, 95%CI = 0.867–1.03, p = 0.171), MMSE (OR = 0.922, 95%CI = 0.784–1.08, p = 0.323), severity of neuropsychiatric symptoms (OR = 1.05, 95%CI = 0.942–1.17, p = 0.378), distress arising from neuropsychiatric symptoms (OR = 0.999, 95%CI = 0.927–1.08, p = 0.974), and clinical dementia rating score (OR = 1.11, 95%CI = 0.877–1.39, p = 0.396). For details, please see Table 2.

**Table 2.** Multivariate logistic regression analysis results.

Variable	Estimate ± SE	Odds ratio	95% CI	*p*
Age (years)	0.009 ± 0.003	1.01	0.961–1.06	0.717
Sex (patient): female^a^	0.527 ± 0.440	1.69	0.715–4.01	0.231
Years of formal education	0.069 ± 0.072	1.07	0.931–1.23	0.336
FAQ	−0.059 ± 0.043	0.943	0.867–1.03	0.171
MMSE	−0.082 ± 0.083	0.922	0.784–1.08	0.323
NPI-S	0.049 ± 0.055	1.05	0.942–1.17	0.378
NPI-D	−0.001 ± 0.038	0.999	0.927–1.08	0.974
CDR-SB	0.100 ± 0.118	1.11	0.877–1.39	0.396

CDR-SB: Clinical Dementia Rating scale Sum of Boxes; CI: confidence interval; FAQ: Functional Assessment Questionnaire; MMSE: Mini-Mental Status Examination; NPI-D: Neuropsychiatric Inventory Distress; NPI-S: Neuropsychiatric Inventory Severity.

^a^ Sex: Reference is male.

### Simple univariate logistic regressions

Simple univariate logistical regression analyses were conducted to further evaluate the extent that the predictors including age, sex, education, FAQ, MMSE, NPI-S, NPI-D, CDR-SB scores, and diagnosis groups can individually predict LTC admission. All independent variables analyzed are included in Table 3.

**Table 3.** Univariate logistic regression analyses results.

Variable	Estimate ± SE	Odds ratio	95% CI	*p*
Age (years)	0.051 ± 0.008	1.05	1.04–1.07	<0.001***
Sex (patient): female^a^	0.584 ± 0.173	1.79	1.28–2.52	<0.001***
Years of formal education	−0.028 ± 0.025	0.973	0.926–1.02	0.271
FAQ	0.082 ± 0.013	1.09	1.06–1.11	<0.001***
MMSE	−0.149 ± 0.021	0.861	0.827–0.897	<0.001***
NPI-S	0.026 ± 0.015	1.03	0.996–1.06	0.093
NPI-D	0.014 ± 0.010	1.01	0.994–1.03	0.171
CDR-SB	0.124 ± 0.032	1.13	1.06–1.21	<0.001***
AD vs. non-AD dementia^b^	−0.282 ± 0.243	0.754	0.468–1.22	0.246

AD: Alzheimer's disease; CDR-SB: Clinical Dementia Rating scale Sum of Boxes; CI: confidence interval; FAQ: Functional Assessment Questionnaire; MMSE: Mini-Mental Status Examination; NPI-D: Neuropsychiatric Inventory Distress; NPI-S: Neuropsychiatric Inventory Severity.

***p < 0.001.

^a^ Sex: Reference is male.

^b^ AD vs. non-AD: reference is those diagnosed with AD.

In a simple logistic regression, age was found to be a significant predictor (OR = 1.05, 95%CI = 1.04–1.07, p < 0.001). This indicates that older age significantly predicted a transition to LTC within two years. Similar results were found for sex, in that being female significantly predicted a transition to LTC (OR = 1.79, 95%CI = 1.28–2.52, p < 0.001). Higher FAQ scores (OR = 1.09, 95%CI = 1.08–1.11, p < 0.001), lower MMSE scores (OR = 0.861, 95%CI = 0.827–0.897, p < 0.001), and higher CDR-SB scores (OR = 1.13, 95%CI = 1.06–1.21, p < 0.001) were all significant.

Some predictors failed to reach statistical significance, including higher education levels (OR = 0.973, 95%CI = 0.926–1.02, p = 0.271), higher NPI-S (OR = 1.03, 95%CI = 0.996–1.06, p = 0.093), and higher NPI-D (OR = 1.01, 95%CI = 0.994–1.03, p = 0.171).

## Discussion

The primary aim of our study was to identify predictors of LTC admission in rural and remote communities in the western Canadian province of Saskatchewan. By identifying these predictors in our patient population, it is possible some risk factors can be targeted with additional interventions or supports to delay the transition to LTC. None of the predictors were significant when entered together in a single model. However, the individual predictors identified include age, sex, FAQ, MMSE, and CDR-SB scores. Being older, female, more functionally dependent, having poorer cognition, and more severe dementia are each associated with a higher risk of LTC placement within two years of initial clinic presentation. This discrepancy should be carefully interpreted but may indicate that each predictor may not have statistically significant unique variance.

The finding that increased age and more severe dementia predict early LTC admission aligns well with previous literature.^7,8,27^ However, Cepoiu-Martin and colleagues found that the effect of increased age diminished after more than two years of follow-up.^27^ While the present study may illustrate predictive value, it is unknown whether this trend would continue with longer follow-up. Meta-analyses and systematic reviews have shown similar results, outlining the robustness of these findings.^6–8^^,27^

Our study found that women also had a higher risk of moving into LTC. This is in stark contrast to a meta-analysis which found that men had a higher risk of institutionalization compared to women.^28^ The underlying reason for this difference may be multifactorial, involving social, demographical, and health-related factors. However, this study can only speculate as to the mechanism behind the heightened risk for women. It may be a demonstration of the “male-female health-survival paradox,” which describes the observation that women typically live longer than men but in turn, have higher morbidity and disability.^29^ Building on the longevity of women compared to men, a study in Germany elucidated a sharp increase in risk of institutionalization when transitioning from marriage to widowhood.^30^ Considering that many informal caregivers tend to be spouses, women with dementia are more likely to lack this spousal support. Another plausible contributor may be that women, although they have a greater cognitive reserve, also have a significantly faster decline in global cognition and executive function compared to men.^28^

A surprising discrepancy was the insignificance of neuropsychiatric symptom severity and caregiver challenges related to neuropsychiatric symptoms, in stark contrast to previous literature.^5,15^ Neuropsychiatric symptoms, including delusions, agitation, depression, and other behavioral disturbances, are frequently cited in literature to be major contributors to the decision to institutionalize.^5–8^ On the contrary, our results indicate that neither the severity of neuropsychiatric symptoms nor the associated caregiver distress are independently significant in the choice to transition to LTC. This was unexpected and raises important questions on the influence that behavioral disturbances have over the transition to LTC in a rural population.

There are a few plausible explanations for this seemingly incongruent finding. Firstly, while neuropsychiatric symptoms undoubtedly affect the caregiver experience, the decision to transition the person with dementia to LTC may be context-dependent and rely more heavily on other metrics besides behavioral disturbances. This may be especially true in the rural and remote context, where access to healthcare services and facilities with the occupancy to accommodate new residents are recognized as barriers in dementia care.^31^ Therefore, we speculate that our findings reflect a reality where these decisions rely on more tangible care demands such as mobility assistance or need for around-the-clock supervision as opposed to neuropsychiatric symptoms.

Secondly, a contributing factor may be the methodology used to measure neuropsychiatric symptom severity. The scales utilized, while both psychometrically validated and reliable, are different. The present study utilized the NPI-D scale, while others used scales such as the Dementia Behavior Disturbance (DBD) scale.^15^ The NPI is considered a more comprehensive and reliable scale in capturing neuropsychiatric symptomatology compared to the DBD scale,^32^ which may explain the difference between studies. However, it is important to acknowledge that the NPI distress scale measures caregiver distress in association with the neuropsychiatric symptoms themselves. Behavioral symptoms undoubtedly contribute to a caregiver's distress. The lack of predictive value in this study may more accurately reflect the complexity behind making a caregiving decision and highlights the multidimensionality behind caregiver distress as a construct when anticipating the transition phase to long-term care.

Identifying these predictors has significant clinical implications for this population. Early identification of higher risk individuals can direct efforts at timely intervention aimed at preserving functional independence as well as cognition. Support programs that target activities of daily living and cognitive preservation strategies would be ideal in this population to potentially delay the LTC transition, in addition to efforts to mitigate caregiver distress. Multicomponent community-based support programs in the past have been shown to reduce caregiver distress, such as the Resources for Enhancing Alzheimer's Caregiver Health (REACH) II program^33^ and another that derives elements from the REACH program and combines it with other community-based supports such as homecare and social workers trained with caregiver transitions.^34^ These programs ultimately use a multimodal approach in formally identifying and meeting caregiver and care recipient needs through skill-based sessions, involvement of professional services, or home visits as required. Although based in the United States, the multicomponent approach appears to have significant effect in not only reducing caregiver distress, but also decreasing frequency and length of hospital visits in care recipients, as well as Center for Epidemiologic Studies Depression Scale scores in care givers and recipients.^33,34^

### Strengths

The most unique strength of this study is the focus on rural and remote populations in comparison to past literature. Saskatchewan has a significant rural population and so, focusing on this group will allow us to tailor and fine-tune our approaches to dementia in rural peoples. A participant count of 635 is also significant, adding power to the statistical analyses. This study has also produced confirmatory results, emphasizing what we know about informal caregiving in the rural context and allowing a foundation for future research (e.g., a qualitative study) to further contextualize how living in a rural community affects their decision-making.

### Limitations

This data was derived from a single center in Saskatchewan only seeing patients from rural and remote communities. Thus, its external validity warrants discussion as we cannot necessarily draw conclusions about other rural and remote populations outside of Saskatchewan. In comparison to their urban counterparts, rural patients and caregivers may have a higher threshold before transitioning to LTC due to lack of nearby facilities or other barriers in accessing healthcare.

A second limitation arises from the retrospective nature of the study. Due to the lack of specific information, we cannot draw conclusions to say that the predictors elucidated are the actual reasons for admission at the individual case level. It is possible that these may be correlated yet unrepresentative of the reasons central to the caregivers’ decisions.

Similarly, a third limitation is the caregiver distress scale. The caregiver distress utilized in this study is specific to the experience of neuropsychiatric symptoms, rather than the holistic caregiver experience. As a result, it is difficult to compare to past literature and may overlook the true predictive value of caregiver distress on the transition to LTC. It is difficult to discern whether the lack of association between neuropsychiatric symptom caregiver distress and long-term care admission may reflect the instrumentation used or of the rural context itself.

### Conclusion

The decision to relocate a person with dementia into long-term care is difficult and requires careful thought and planning in navigating an emotionally charged and difficult transition. Our study describes the unique rural and remote Saskatchewan population and identifies the factors that affects our population's decision to transition into long-term care. As this clinic continues to provide care to our rural population, these results direct our intervention strategies and allow us to refine our approach to caring for rural persons with dementia.
